# PulmoBind Imaging Measures Reduction of Vascular Adrenomedullin Receptor Activity with Lack of effect of Sildenafil in Pulmonary Hypertension

**DOI:** 10.1038/s41598-019-43225-3

**Published:** 2019-04-29

**Authors:** Nassiba Merabet, Mohamed Jalloul Nsaibia, Quang Trinh Nguyen, Yan Fen Shi, Myriam Letourneau, Alain Fournier, Jean-Claude Tardif, François Harel, Jocelyn Dupuis

**Affiliations:** 1 Research Center, Montreal Heart Institute, 5000, Belanger, Montreal, QC H1T 1C8 Canada; 2 Department of Medicine, Université de Montréal, Montréal, Québec Canada; 3 Department of Nuclear Medicine and radiology, Université de Montréal, Montréal, Québec Canada; 4 INRS-Institut Armand Frappier, Laval, Québec Canada

**Keywords:** Preclinical research, Experimental models of disease, Vascular diseases, Diagnostic markers, Molecular imaging

## Abstract

Endothelial dysfunction is a core pathophysiologic process in pulmonary arterial hypertension (PAH). We developed PulmoBind (PB), a novel imaging biomarker of the pulmonary vascular endothelium. ^99m^Technetium (^99m^Tc)-labelled PB binds to adrenomedullin receptors (AM_1_) densely expressed in the endothelium of alveolar capillaries. We evaluated the effect of sildenafil on AM_1_ receptors activity using ^99m^Tc-PB. PAH was induced in rats using the Sugen/hypoxia model and after 3 weeks, animals were allocated to sildenafil (25 or 100 mg/kg/day) for 4 weeks. ^99m^Tc-PB uptake kinetics was assessed by single-photon emission computed tomography. PAH caused right ventricular (RV) hypertrophy that was decreased by low and high sildenafil doses. Sildenafil low and high dose also improved RV function measured from the tricuspid annulus plane systolic excursion. Mean integrated pulmonary uptake of ^99m^Tc-PB was reduced in PAH (508% · min ± 37, p < 0.05) compared to controls (630% · min ± 30), but unchanged by sildenafil at low and high doses. Lung tissue expressions of the AM_1_ receptor components were reduced in PAH and also unaffected by sildenafil. In experimental angio-proliferative PAH, sildenafil improves RV dysfunction and remodeling, but does not modify pulmonary vascular endothelium dysfunction assessed by the adrenomedullin receptor ligand ^99m^Tc-PB.

## Introduction

Pulmonary arterial hypertension (PAH), or group I pulmonary hypertension (PH), is a rare but fatal disease characterized by progressive elevation in pulmonary artery pressure. Pathophysiologically, it is caused by dysfunction of the endothelium leading to proliferation of vascular wall cells and progressive blockage of pulmonary arterioles. PAH is a complication of various diseases that leads, in its advanced stages, to impaired right ventricular (RV) function and ultimately death.

Currently, nuclear imaging of the lung vessels is performed by using ^99m^Technetium-labelled macroaggregates of albumin (^99m^Tc-MAAs). Macroaggregates are retained within the pulmonary arterioles in relation to their diameter (50–150 μm) and in proportion to the distribution of flow. Consequently, this “physical” tracer cannot diagnose non-anatomical abnormalities associated with pulmonary vascular disease (PVD) and its capacity to reveal small resistance arteries obstruction is limited by the size of the particles and by spatial resolution. This is particularly relevant to PAH that affects lung arterioles and explains why human subjects with PAH can have apparently normal ^99m^Tc-MAAs lung perfusion scans. Additionally, ^99m^Tc-MAAs obstruct small arteries in subjects with an already reduced lung perfusion with inherent risks associated with the use of a human-derived product. PulmoBind (PB) is a novel molecular SPECT imaging agent that distributes according to the lung capillaries perfusion by binding to the vascular endothelium^1–6^. PB is a specific ligand of the adrenomedullin receptor (AM_1_). PB was specifically developed for molecular SPECT imaging of the pulmonary circulation by labelling with ^99m^Tc. AM_1_ receptors, a dimer composed of the calcitonin like receptor (CLR) and the receptor activity modifying protein-2 (RAMP2)^7^, are densely expressed on the endothelial surface of human alveolar capillaries^8,9^ where they mediate the predominant lung clearance of this peptide^10^. A safety and dosimetric phase I study of PB in humans was recently completed^6^, as well as a phase II study in 30 patients with group I and group IV PH^4^. PB was found to be safe with rapid and important lung uptake providing excellent quality imaging. In patients with PH, ^99m^Tc-PB showed severe abnormalities with heterogenous “drop zones” where there was no uptake of the tracer.

The currently approved treatments of PAH target endothelial dysfunction. The vasodilatory effects of NO are caused by the second messenger cyclic guanosine monophosphate (cGMP) that is promptly hydrolysed by phosphodiesterases (PDE). PDE type 5 (PDE-5) is the predominant lung isoform and is up-regulated in pathologies causing PAH. Sildenafil, a selective PDE-5 inhibitor, leads to intracellular accumulation of cGMP and therefore augments NO-mediated vasodilatation. Sildenafil has also been shown to have antiproliferative effects on pulmonary vascular smooth-muscle cells. Studies involving animal models and patients with PAH suggest that sildenafil is beneficial in the treatment of PAH. It is however still unclear whether the effects are primarily mediated by a vasodilatory action with secondary improvement in RV function or whether sildenafil can directly impact on the pathophysiologic process of PVD.

The Sugen/hypoxia model (SUHx) of angio-proliferative PAH presents pathologic morphological lesions of PVD similar to those observed in human PAH^11^. Therefore, the primary aims of this study were: (1) to evaluate the capacity of the endothelial cell tracer ^99m^Tc-PB to detect the presence of angio-proliferative PVD by imaging the AM_1_ receptor in the SUHx of PAH and (2) to investigate the potential therapeutic effects of two doses of sildenafil on angio-proliferative PVD by assessing ^99m^Tc-PB uptake kinetics.

## Methods

All experimental procedures were performed in accordance with regulations and ethical guidelines from the Canadian Council for the Care of Laboratory Animals and received approval by the animal ethics and research committee of the Montreal Heart Institute. Adult male Sprague-Dawley rats with a body weight of 150–200 g were obtained from Charles River Laboratories. After a single subcutaneous injection of the vascular endothelial growth factor receptor blocker Sugen 5416 (SU) (20 mg/kg), rats were exposed to chronic hypobaric hypoxia (Hx) (10% oxygen) for 3 weeks. They were then returned to normoxia for 4 weeks. All experiments were therefore performed 7 weeks after SU injection. Some Animals were treated by once-daily gavage with sildenafil citrate (SI180-0100; Galenova, Quebec, Canada) at a dose of 25 mg/kg/day or 100 mg/kg/day every morning. Therapy was started after the 3 weeks of hypoxia for a duration of four weeks and was not administered on the morning of the experiments.

### Transthoracic Echocardiographic Study

Examinations were performed using a phased-array probe 10S (4.5–11.5 Megahertz) and Vivid 7 Dimension ultrasound system (GE Healthcare Ultrasound, Horten, Norway), with rats being sedated by (1.5–2%) isofluorane. Left ventricular (LV) dimension at end-diastole (LVDd) was measured by M-mode echocardiography. M-mode was also used to measure RV dimension and RV anterior wall thickness at end-diastole (RVDd, RVAWd), as well as tricuspid annulus plane systolic excursion (TAPSE). Pulmonary acceleration time (PAAT) was measured by pulmonary artery flow recorded by Pulsed wave Doppler (PW). The average of 3 consecutive cardiac cycles was used for each measurement, with the operator being blinded to treatment assignment.

### *In Vivo* Biodistribution and Lung Uptake of ^99m^Tc-PB

Animals were anesthetized by isoflurane (1.5–2%). ^99m^Tc-labeled PB (37–58 MBq) was prepared as previously described in details^6^ and intravenously injected in a volume of 300 μL via the caudal vein. *In vivo* whole-body biodistribution of radioactivity was measured by a nuclear medicine camera (Ecam, Siemens) equipped with an on-board computer and a low-energy high resolution parallel-hole collimator. Dynamic acquisitions were recorded for a 60-min period, and static whole-body scans were obtained at 30 min and 60 min after injection. Dynamic and static acquisitions were evaluated with MATLAB version 7.01 image analysis tool software. Results were expressed as the percentage of the injected dose (%ID).

### Pathologic assessment of right ventricular hypertrophy

After excision of the heart, the RV wall was separated from the LV wall and the interventricular septum. The ratio of the RV to LV plus septum [RV/LV + S] weight was calculated as an index of RV hypertrophy (RVH).

### Adrenomedullin Receptor Expression in lung tissue by quantitative real-time PCR

RNA was extracted from homogenized lung tissue using the Qiagen TRIzol reagent (Fisher Scientific) and treated with TURBO DNA-free DNase (Fisher Scientific) as per manufacturer’s instructions. Extracted RNA was converted to cDNA using GoScript Reverse Transcriptase with 500–1000 ng starting material per reaction. The quality of total RNA was monitored by capillary electrophoresis (Experion, Biorad, ON, Canada). Quantitative real-time PCR (qPCR) was performed with QuantiTect SYBR Green PCR kit (from Qiagen, CA) on the Mx3005P real-time PCR machine (Stratagene, CA). Primers for RAMP2 and CLR (rat) were obtained from Qiagen (ON, Canada). PCR data was analyzed using software MxPro (Stratagene, CA). Comparative quantitative analysis was performed based on a delta-delta Ct (ΔΔCt) method using HPRT1 gene (Life technologies/Thermo Fisher Scientific,ON, Canada) as normalization controls.

### Western immunoBlots for AM_1_ receptors expression in lungs

To perform lung protein extraction, the snap-frozen right inferior lobe was homogenized using a polytron homogenizer in lysis buffer containing a protease inhibitor cocktail followed by centrifugation, supernatants were harvested, and protein loading buffer was added. Samples were boiled 5 minutes, 20 µg of proteins were loaded onto polyacrylamide gels (15% SDS-PAGE) followed by electrophoresis and transferred onto nitrocellulose membranes. Membranes were blocked with TBS-tween containing 5% non-fat dry milk or 5% BSA, according to manufacturer’s instructions, incubated with either RAMP2 (1:500) (from Santa Cruz Biotechnologies, TX, USA) or β-ACTIN (1:1000) (from Sigma-Aldrich, ON, Canada) primary antibodies overnight at 4 °C. Membranes were then washed and incubated with HRP-labeled secondary antibodies (1: 10,000) (from Cell Signaling Technology, MA, USA). Detection was done using clarity western ECL substrate (BioRad, ON, Canada). Images were acquired, and quantification analyses were performed using a ChemiDocMP system (BioRad, ON, Canada) and densitometric analyses of Western blot were performed using ImageLab version 5.2.1 (Bio-Rad).

### Statistical analyses

Differences between groups were evaluated by ANOVA followed, when a significant interaction was found, by Tukey’s multiple comparisons. For molecular biology experiments (qPCR and western blots), respective groups were compared by using 2-tailed independent sample t-tests. Analysis was performed using Prism software (version 6.0; GraphPad). All results are given as mean ± SEM. Differences were considered significant at the level *p* < *0*.*05*.

## Results

**Figure 1:**
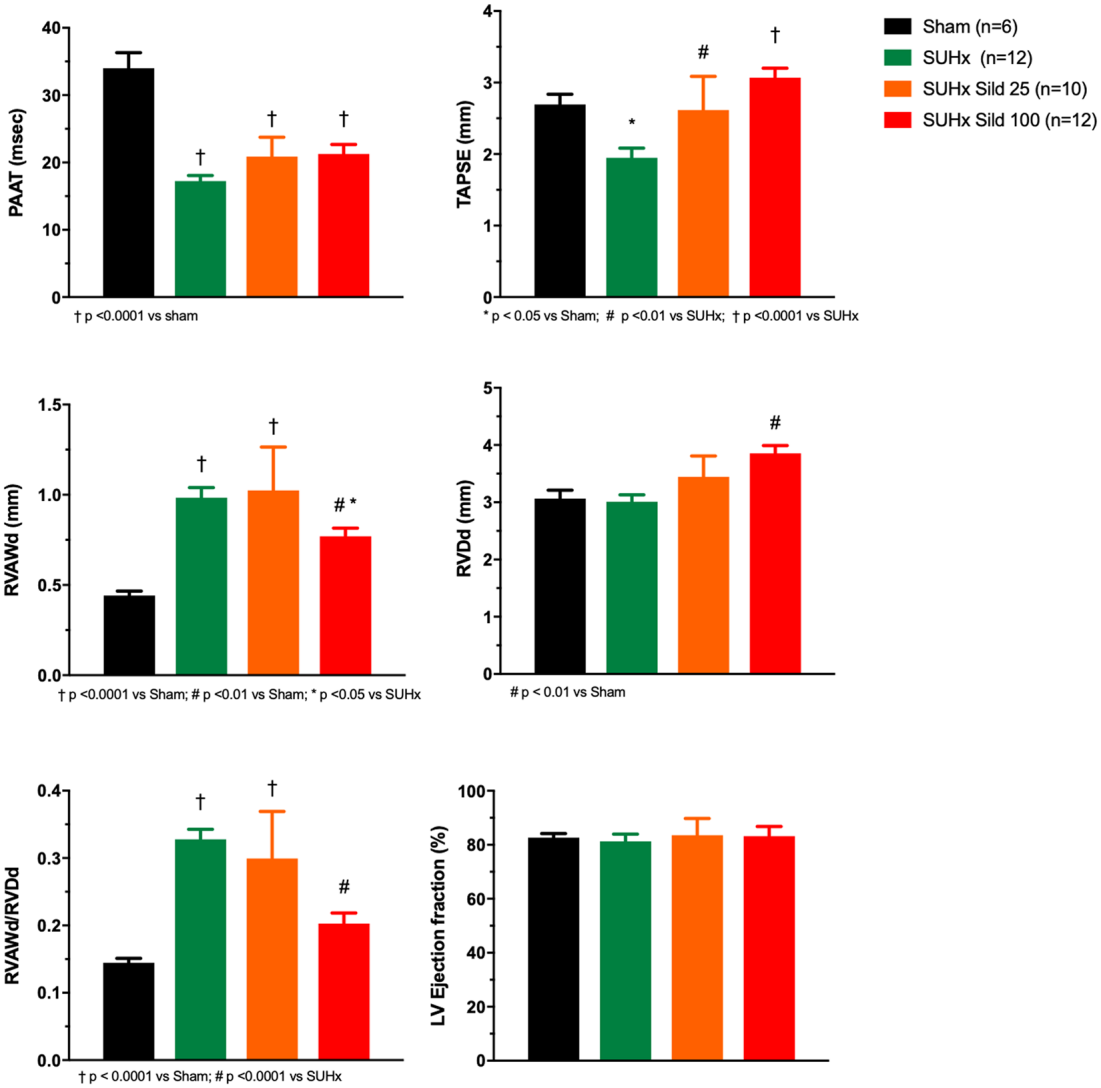
Echocardiographic parameters. Pulmonary artery acceleration time (PAAT), tricuspid annulus plane systolic excursion (TAPSE), right ventricular anterior wall thickness (RVAWd), right ventricular diastolic diameter (RVDd), remodeling index (RVAWd/RVDd), left ventricular (LV). Sugen-Hypoxia induced pulmonary hypertension (SUHx). Treatment with sildenafil 25 mg/kg/day (Sild 25) and 100 mg/kg/day (Sild 100). Differences between groups evaluated by ANOVA followed by Tukey’s multiple comparisons.

**Figure 2:**
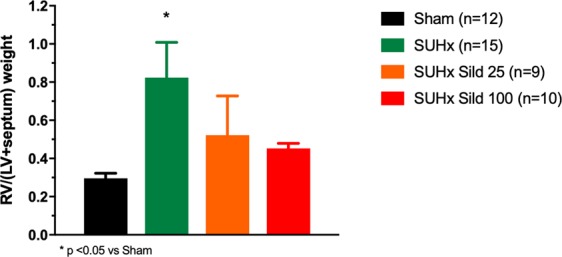
Right ventricular hypertrophy. Ratio of the right ventricular weight over the left ventricular plus septum weight (RV/(LV + septum)). Sugen-Hypoxia induced pulmonary hypertension (SUHx). Treatment with sildenafil 25 mg/kg/day (Sild 25) and 100 mg/kg/day (Sild 100). Differences between groups evaluated by ANOVA followed by Tukey’s multiple comparisons.

Untreated animals developed severe PAH, RVH and RV dysfunction as evaluated from echocardiography (Fig. 1). PAAT, a parameter strongly inversely correlated with pulmonary artery systolic pressure, was markedly reduced. This was mildly but non-significantly improved by sildenafil therapy. RV function as evaluated from the tricuspid annular plane systolic excursion (TAPSE), was reduced and dose-dependently improved by sildenafil. There was also evidence of RV remodeling with increased thickness of the anterior RV wall (RVAWd) that was improved by the higher dose sildenafil. There was mild increase in RV diastolic diameter (RVDd) only in the higher dose sildenafil group. The RV remodeling index, measured as the ratio of thickness over diameter (RVAWd/RVDd) was markedly increased and improved by the higher dose of sildenafil. There was no difference in LV ejection fraction between groups. Pathological RVH was confirmed by the Fulton index showing severe RVH in the SUHx animals. This was reduced and no longer different from the sham group at both dosages of sildenafil (Fig. 2).

**Figure 3:**
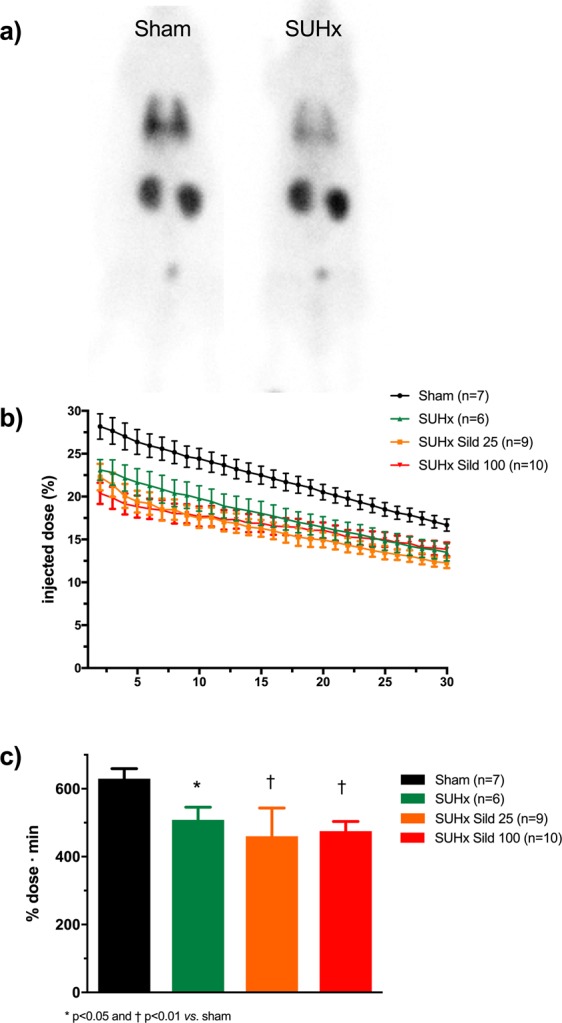
Lung vascular imaging with ^99m^Tc-PulmoBind. (**a**) Example of whole body planar imaging of a sham rat and PAH rat 5 minutes after tail vein injection in a 5 minutes acquisition. (**b**) Pulmonary ^99m^Tc-PulmoBind activity over 30 minutes. (**c**) Integrated pulmonary ^99m^Tc-PulmoBind uptake. Differences between groups evaluated by ANOVA followed by Tukey’s multiple comparisons.

An example of whole-body ^99m^Tc-PulmoBind lung scan in a control rat and a SUHx rat is shown in Fig. 3a, 5 minutes after tail vein injection. Predominant lung uptake of the tracer is seen with also some activity in the kidneys. In the SUHx rat, uptake was homogeneously reduced. A video demonstrating the dynamic uptake of ^99m^Tc-PB over 30 minutes is available as supplemental material (Supplement Video S1). Tomographic imaging of lungs (Supplement Fig. S1) and a video demonstrating 360 degrees tomographic imaging (Supplement Video S2) are also presented as supplements. The time course of lung ^99m^Tc-PB activity over 30 minutes demonstrates peak uptake of about 30% injected followed by slow decay of activity down to about 20% after 30 minutes (Fig. 3b). The SUHx PAH animals showed lower ^99m^Tc-PB uptake at all time points with no effect of sildenafil therapy at both low and higher dosages. The integrated lung activity over 30 minutes was lower in the PAH animals, with no effect of therapy (Fig. 3c).

**Figure 4:**
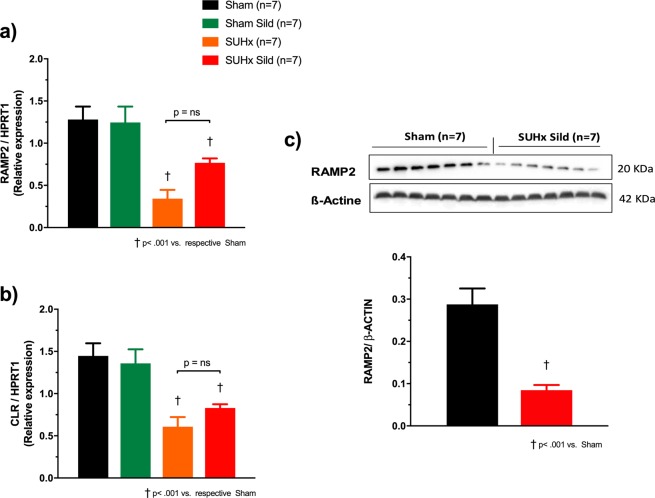
Lung adrenomedullin (AM_1_) receptor expression and effect of sildenafil (100 mg/kg/day). Expression of mRNA by qPCR of the dimeric components of the AM_1_ receptor; (**a**) receptor activity modifying protein-2 (RAMP2) and (**b**) calcitonin-like receptor (CLR). (**c**) Protein expression of RAMP2. Respective groups were compared using 2-tailed independent sample t-tests.

The message expression of the dimeric components of the specific AM_1_ receptor was quantified in whole lung tissues by qPCR. Both the receptor activity modifying protein-2 (RAMP2) and the calcitonin like-receptor (CLR) were markedly reduced in the lungs of PAH animals (Fig. 4). High-dose sildenafil did not significantly affect the receptors in both sham and PAH animals. Protein expression of RAMP2, the distinguishing component of the AM_1_ receptor was similarly reduced in sildenafil-treated SUHx rats.

## Discussion

The main objectives of this study were: (1) to evaluate the capacity of the endothelial cell tracer ^99m^Tc-PB to detect the presence of angio-proliferative PVD by imaging the AM_1_ receptor in the SUHx model of PAH and (2) to investigate the potential therapeutic effects of sildenafil on angio-proliferative PVD by assessing ^99m^Tc-PB uptake kinetics. We found that pulmonary uptake of ^99m^Tc-PB was reduced in this angio-proliferative model of PAH and that this marker of PVD was not affected by sildenafil therapy, despite evidence for improvement of RV function and remodeling.

Combined use of the VEGF receptor antagonist Sugen 5416 and exposure to hypoxia in rats leads to severe PAH closely mimicking apoptotic-dependent and angio-proliferative PAH seen in humans^11^. In PAH, endothelial and smooth muscle cells proliferations cause progressive obliteration of pulmonary arterioles leading to PH, RVH, RV dysfunction and ultimately death. Unsurprisingly, we accordingly found that 7 weeks after SUHx exposure (including 3 weeks of hypoxia), rats developed severe PAH with markedly reduced PAAT and severe RVH and RV dysfunction. Cardiac ultrasound measured PAAT, a parameter closely indirectly related to pulmonary artery systolic pressure in rat models of PH^12,13^, was markedly reduced in SUHx. Sildenafil therapy mildly but non-significantly increased PAAT. However, it significantly reduced RVH both pathologically (RV/(LV + septum) weight) and at cardiac ultrasound (RVAWd), while it also improved RV function (TAPSE). The effects of sildenafil on RVH and RV function, but not on PAAT, were dose-dependent with evidence of greater benefit at the higher dosage. These data therefore suggest a marked effect of sildenafil on RV function and remodeling. This finding is consistent with previous studies showing that the ability of PDE-5 inhibitors to increase RV inotropy and to decrease RV afterload, without significantly affecting systemic hemodynamics, makes them suitable for the treatment of PAH^14^. There is indeed an increase in RV PDE-5 expression in patients and animals with PAH^14^.

The current study did not directly evaluate the effects of sildenafil on histological pulmonary vascular remodeling. Another limitation is that we did not measure invasive pulmonary hemodynamics so as to avoid interference with the imaging protocol. Acutely administered sildenafil either orally, intratracheally or intravenously, is an effective pulmonary vasodilator^15^ in this model, while mixed results were however reported on pulmonary vascular remodeling with chronic administration. Sildenafil was administered to rats at 50 mg/kg twice daily following two weeks of hypoxia for a duration of three weeks^16^. Although this 5-week protocol resulted in less severe PAH than in our study (a 7-week protocol), sildenafil also effectively limited RVH measured by Fulton’s index, but did not reduce the proportion of pulmonary arterioles obliteration and the number of plexiform lesions and did not reduce PH severity^16^. These authors therefore concluded that these observations suggest that sildenafil prevented the hyperplasia and hypertrophy caused by sustained contraction due to vasodilatory effects, but did not alter the course of the arteriolar disease. However, other investigators found that oral sildenafil at 50 mg/kg once daily for 14 days did provide positive pulmonary vascular remodeling with reduced percentage of muscularization, reduced medial hypertrophy and reduced number of occluded small arteries^17^. More recently, beneficial arteriolar remodeling was also present with chronic inhaled sildenafil in short or long acting formulations in the same model^18^. Adrenomedullin (AM) is a 52 amino acids peptide with vasodilator and anti-proliferative actions. AM is a pulmonary vasodilator^19^ and promotes lung angiogenesis and alveolar development and repair^20^. Chronic AM infusion reduces PH and RVH in the rat monocrotaline model of PAH^21^. The specific AM receptor (AM_1_), a heterodimer composed of CLR and RAMP2, is densely distributed on the vascular endothelium of alveolar capillaries^8,9^. Accordingly, AM is rapidly extracted upon its first passage through the pulmonary circulation^4,6,10^. PB is a molecular tracer derived from human AM that was specifically engineered to bind to the AM_1_ receptor to detect PVD^1^. ^99m^Tc-PB was successfully used for the imaging of the normal pulmonary circulation in animals and humans and we previously confirmed the ability of this molecular tracer to detect PVD associated with PAH^6,22^. We demonstrated that ^99m^Tc-PB can diagnose large perfusion defects associated with pulmonary embolism as well as pulmonary microvascular disease^22^. In the rat monocrotaline model of PAH, ^99m^Tc-PB uptake is markedly reduced with a reduction of RAMP2 lung expression^22^. In human PH, a recent phase II study showed that PulmoBind could detect large defects in patients with unoperated chronic thromboembolic PH, as well as smaller heterogenous defects or “drop out” zones in subjects with group I PH^4^. The current study was a first attempt at evaluating the effect of PAH specific therapy on lung ^99m^Tc-PB uptake.

Similar to the monocrotaline model, we confirm the ability of ^99m^Tc-PB to detect PVD in the SUHx model of PAH. The reduction was visually homogeneous, confirming the diffuse PVD distribution in this model. Interestingly, sildenafil did not modify ^99m^Tc-PB uptake. Reduced ^99m^Tc-PB uptake can be caused by a diminished expression of the AM_1_ receptor, a reduced accessibility to the receptor following microvascular obliteration or a combination of both. Our analysis does not allow distinction between these two mechanisms but since we found reduced expression of CLR and RAMP2, this suggests that a combination of both is operative in this model as in the monocrotaline model. Since previous studies showed mixed results on the effects of sildenafil on pulmonary vascular obliteration in this model, our findings suggest that sildenafil therapy does not significantly modify the metabolically active pulmonary vascular surface available for binding of circulating ^99m^Tc-PB. The exact pathophysiologic significance of this finding remains to be determined and whether different sildenafil dosing regimens and other PAH therapeutic agents could improve ^99m^Tc-PB uptake need to be tested.

The current study demonstrates the feasibility and potential utility of molecular imaging of PVD. This exciting field of research may provide personalized information on the status of PVD as agents assessing different pathophysiologic processes are being developed. A recent study used 3′-Deoxy-3′-[18 F]Fluorothymidine positron emission tomography (PET) in human PAH and animal models to demonstrate increased lung cellular proliferation^23^. We also recently successfully developed a NOTA-derivatized adrenomedullin analog (DFH17) radiolabeled with [(18)F]AlF for PET imaging of pulmonary microcirculation^24^ that would provide greater temporal and spatial resolution compared to ^99m^Tc-PB.

## Conclusion

We used the AM_1_ receptor ligand ^99m^Tc-PB to image and detect pathologic variations of pulmonary microcirculation in an apoptosis-dependent angio-proliferative PAH model. Pulmonary ^99m^Tc-PB uptake was reduced by PAH and unaffected by sildenafil therapy. Whether other therapies could successfully modulate this endothelial dysfunction associated with PAH will require further studies but may prove useful to potentially detect PVD modifying agents.

## Supplementary information

Supplementary information

Supplement video S1

Supplement video S2

## References

[1] Letourneau M, Nguyen QT, Harel F, Fournier A, Dupuis J (2013). PulmoBind, an adrenomedullin-based molecular lung imaging tool. J Nucl Med.

[2] Dupuis J, Harel F, Nguyen QT (2014). Molecular imaging of the pulmonary circulation in health and disease. Clin Transl Imaging.

[3] Levac X (2016). Evaluation of pulmonary perfusion by SPECT imaging using an endothelial cell tracer in supine humans and dogs. EJNMMI research.

[4] Harel F (2017). Molecular imaging of the human pulmonary vascular endothelium in pulmonary hypertension: a phase II safety and proof of principle trial. European journal of nuclear medicine and molecular imaging.

[5] Harel F (2008). Use of Adrenomedullin Derivatives for Molecular Imaging of Pulmonary Circulation. Journal of Nuclear Medicine.

[6] Harel F (2015). Molecular imaging of the human pulmonary vascular endothelium using an adrenomedullin receptor ligand. Mol Imaging.

[7] Hay DL, Garelja ML, Poyner DR, Walker CS (2018). Update on the pharmacology of calcitonin/CGRP family of peptides: IUPHAR Review 25. Br J Pharmacol.

[8] Hagner S, Stahl U, Knoblauch B, McGregor GP, Lang RE (2002). Calcitonin receptor-like receptor: identification and distribution in human peripheral tissues. Cell Tissue Res.

[9] Hagner S (2003). Immunohistochemical detection of the calcitonin receptor-like receptor protein in the microvasculature of rat endothelium. Eur J Pharmacol.

[10] Dupuis J, Caron A, Ruel N (2005). Biodistribution, plasma kinetics and quantification of single-pass pulmonary clearance of adrenomedullin. Clin Sci (Lond).

[11] Nicolls MR (2012). New models of pulmonary hypertension based on VEGF receptor blockade-induced endothelial cell apoptosis. Pulm Circ.

[12] Jones JE (2002). Serial noninvasive assessment of progressive pulmonary hypertension in a rat model. Am J Physiol Heart Circ Physiol.

[13] Dayeh NR (2018). Echocardiographic validation of pulmonary hypertension due to heart failure with reduced ejection fraction in mice. Sci Rep.

[14] Nagendran J (2007). Phosphodiesterase type 5 is highly expressed in the hypertrophied human right ventricle, and acute inhibition of phosphodiesterase type 5 improves contractility. Circulation.

[15] Rashid J (2017). Inhaled sildenafil as an alternative to oral sildenafil in the treatment of pulmonary arterial hypertension (PAH). J Control Release.

[16] Bhat L (2017). RP5063, a novel, multimodal, serotonin receptor modulator, prevents Sugen 5416-hypoxia-induced pulmonary arterial hypertension in rats. Eur J Pharmacol.

[17] Lang M (2012). The soluble guanylate cyclase stimulator riociguat ameliorates pulmonary hypertension induced by hypoxia and SU5416 in rats. PLoS One.

[18] Rashid J, Nozik-Grayck E, McMurtry IF, Stenmark KR, Ahsan F (2019). Inhaled combination of sildenafil and rosiglitazone improves pulmonary hemodynamics, cardiac function, and arterial remodeling. Am J Physiol Lung Cell Mol Physiol.

[19] Nagaya N (2000). Haemodynamic and hormonal effects of adrenomedullin in patients with pulmonary hypertension. Heart.

[20] Vadivel A (2010). Adrenomedullin promotes lung angiogenesis, alveolar development, and repair. Am J Respir Cell Mol Biol.

[21] Yoshihara F (1998). Chronic infusion of adrenomedullin reduces pulmonary hypertension and lessens right ventricular hypertrophy in rats administered monocrotaline. Eur J Pharmacol.

[22] Dupuis J (2009). Molecular imaging of monocrotaline-induced pulmonary vascular disease with radiolabeled linear adrenomedullin. J Nucl Med.

[23] Zhao L (2013). Heterogeneity in lung (18)FDG uptake in pulmonary arterial hypertension: potential of dynamic (18)FDG positron emission tomography with kinetic analysis as a bridging biomarker for pulmonary vascular remodeling targeted treatments. Circulation.

[24] Alonso Martinez LM (2018). Al[(18)F]F-complexation of DFH17, a NOTA-conjugated adrenomedullin analog, for PET imaging of pulmonary circulation. Nucl Med Biol.

