# Supplementary material for: PulmoBind Imaging Measures Reduction of Vascular Adrenomedullin Receptor Activity with Lack of effect of Sildenafil in Pulmonary Hypertension
Source: Sci Rep. 2019 Apr 29;9:6609. doi: 10.1038/s41598-019-43225-3 (PMC6488585; doi:10.1038/s41598-019-43225-3)
Supplement: Supplementary file 1 — Supplementary information [file 41598_2019_43225_MOESM1_ESM.pdf]

PulmoBind Imaging Measures Reduction of Vascular Adrenomedullin Receptor Activity with Lack of effect of Sildenafil in Pulmonary Hypertension

Nassiba Merabet[1], Mohamed Jalloul Nsaibia[1], Quang Trinh Nguyen[1], Yan Fen Shi[1], Myriam Letourneau[4], Alain Fournier[4], Jean-Claude Tardif[1,2], François Harel[1,3], *Jocelyn Dupuis[1,2].

1. Research Center, Montreal Heart Institute, 5000, Belanger, Montreal, QC H1T 1C8, Canada.

2. Department of Medicine, Université de Montréal, Montréal, Québec, Canada.

3. Department of Nuclear Medicine and radiology, Université de Montréal, Montréal, Québec, Canada.

4. INRS-Institut Armand Frappier, Laval, Québec, Canada

Supplementary information

Supplement figure S1. Example of SPECT imaging of rat lungs with ^{99m}Tc-PulmoBind in coronal, sagittal and axial views.

Supplement video S1. Video of whole body distribution of ^{99m}Tc-PulmoBind following tail vein injection in a rat.

Supplement video S2. Video reconstruction (360 degrees) SPECT imaging of rat lungs with ^{99m}Tc-PulmoBind.

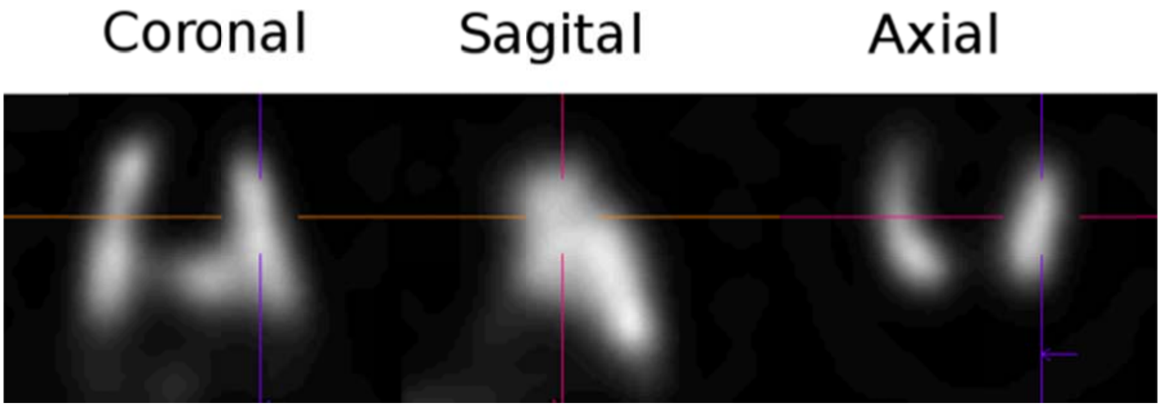

Supplemental figure S1
